# HIV-AIDS epidemiological model in Constanța County

**DOI:** 10.1186/1471-2334-14-S7-P36

**Published:** 2014-10-15

**Authors:** Iulia Gabriela Șerban, Sorin Rugină

**Affiliations:** 1 Regional HIV/AIDS Center Constanța, Romania; 2 Faculty of Medicine, “Ovidius” University, Constanța, Romania

## Background

The evolution of AIDS cases in Constanța County during 1985-2003 shows a rapid upward detection curve in children in the first decade, a low rate of screening in adults in the first decade, with absolute predominance of cases in children and progressively slower increase in the number of cases identified in the second decade, on account of the increase in the number of cases in adults. The main objective of this paper is to present the retrospective and prospective HIV-AIDS epidemiological model.

## Methods

Organization and data collection was performed, through analysis in terms of ordering data, correlation, and validation: descriptive analysis of the elements that determine the distribution of cases. We have identified characteristics from: 1. Time - tendency (age pyramid); 2. Place - geographic (latitude, longitude - Black Sea port, airport, tourist city), geological, industrial (petrochemical - Midia Năvodari), agriculture, climatic, geopolitical conditions, prior to 1989, blood products, pollution status, place of work (childcare institutions, sex workers, accidental exposure); 3. We evaluated the determinants of public health problems - variable 'person' (belonging to a group of high-risk, the health providers, travelers, isolated populations, risk of infection). Aspects of demography - biological epidemiological indicators like gender/sex [M, F], age [age group, adult - children], ethnicity, race, area of origin [U, R], village of origin, place of residence, behaviors [donor blood, MSM, heterosexual, IDU, sex workers], profession, occupation, life style, socio-economic level, and other information - migration, tourism) We proposed to observe, to describe cumulative data, the explanation of the phenomenon, to monitor, to control (preventive measures, and curative - ARV) and to formulate the considerations based on epidemiological verified data.

## Results

In Constanța, the top of the curve-infection was 1988, possible transfusion (epidemiological surveys). Constanța - first certified center in Romania, first family foster homes and palliative therapy center, the highest percentage (99%) of testing of pregnant women. Healthcare system in Constanța always has anticipated the national Healthcare System development (epidemiological information flow models). The tracking system through clinical indicators to monitor and evaluate (July 2009). Initially management of a pioneer public-private partner relationship with Baylor Constanța is methodological center.

## Conclusion

The role of epidemiologist has been required - the clinician community! The role of the multidisciplinary team is relevant. Establishment of epidemiological valuable databases on the environmental impact (transmission, iatrogenic) of HIV can be a model for monitoring other chronic infections (hematology, hepatology, and oncology).

